# Pathophysiology-driven neuroimaging: decoding brain injury in sepsis

**DOI:** 10.3389/fnmol.2026.1840888

**Published:** 2026-06-15

**Authors:** Rui Yan, Guanghui Ma, Haixia Huang, Jinyuan Mei, Qizhang Yang, Ye Gong, Mi Tian

**Affiliations:** 1 Department of Critical Care Medicine, Huashan Hospital, Fudan University, Shanghai, China; 2 Department of Radiology, Shanghai Health and Medical Center, Wuxi, Jiangsu, China; 3 Department of Neurosurgery, Huashan Hospital, Fudan University, Shanghai, China

**Keywords:** blood–brain barrier, neuroimaging, neuroinflammation, sepsis, sepsis-associated encephalopathy

## Abstract

Sepsis-associated encephalopathy (SAE) is a diffuse brain dysfunction induced by sepsis, characterized by high mortality and frequent long-term cognitive impairment. Its pathogenesis involves multiple mechanisms, including endothelial activation, blood-brain barrier (BBB) disruption, neuroinflammation, and neurotransmitter imbalance, ultimately resulting in neuronal and glial injury. Recent advances in neuroimaging have provided new insights into the pathological features of SAE, while offering valuable tools for early diagnosis, prognostic stratification, and the identification of potential therapeutic targets. This Review summarizes recent advances in neuroimaging research in SAE, integrating clinical findings with pathophysiological and experimental evidence to delineate imaging phenotypes, elucidate underlying mechanisms, and highlight the translational potential of emerging imaging modalities.

## Graphical Abstract

**Figure:**
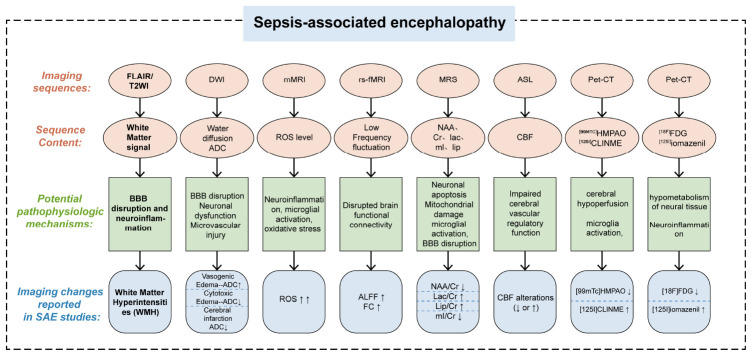
Multimodal neuroimaging markers and pathophysiological correlates in sepsis-associated encephalopathy. Graphical Abstract Summary of multimodal neuroimaging techniques used in sepsis-associated encephalopathy (SAE), the biological/pathophysiological processes they reflect, and their typical imaging readouts. DWI primarily reflects water diffusion/ADC changes and may indicate BBB disruption, neuronal dysfunction, microvascular injury, vasogenic/cytotoxic edema, or cerebral infarction. mMRI can assess reactive oxygen species (ROS)-related changes and is linked to neuroinflammation, microglial activation, and oxidative stress. rs-fMRI captures low-frequency fluctuations and functional connectivity (e.g., ALFF and FC), reflecting disrupted brain network function. MRS evaluates metabolic biomarkers (e.g., NAA, Cr, lactate, myo-inositol, and lipids), which may indicate neuronal apoptosis, mitochondrial injury, microglial activation, and BBB dysfunction. ASL measures CBF and may reveal impaired cerebrovascular autoregulation and perfusion abnormalities. PET-CT (e.g., [99mTc] HMPAO and [125I] CLINME-related signals, depending on study design) provides molecular information on cerebral hypoperfusion and glial activation. This multimodal framework highlights the complementary value of structural, functional, metabolic, and molecular imaging for understanding SAE.

##  1 Introduction

Sepsis is a leading cause of death and long-term disability among patients in the intensive care unit (ICU) and is characterized by life-threatening organ dysfunction resulting from a dysregulated host response to infection (Singer et al., 2016). Among the multiple organ systems affected, the brain is particularly vulnerable, often exhibiting early signs of injury (Angus et al., 2001). This diffuse dysfunction, caused by systemic rather than direct central nervous system (CNS) infection, is termed SAE (Sonneville et al., 2023). Due to the lack of unified diagnostic criteria, the reported incidence of SAE varies widely across studies, ranging from 9% to 71% (Molnár et al., 2018). SAE is closely associated with adverse clinical outcomes, including increased mortality (Angus et al., 2001; Schuler et al., 2018), persistent cognitive impairment among survivors (Iwashyna et al., 2010; Prescott and Angus, 2018), and a higher burden of neuropsychiatric sequelae, including depression, anxiety, and post-traumatic stress disorder. Early recognition remains challenging in ICU settings, increasing the need for complementary diagnostic approaches (Gofton and Young, 2012; Dumbuya et al., 2023).

Neuroimaging offers complementary structural, diffusion, perfusion, metabolic, and functional information in SAE. Accordingly, this Review summarizes current clinical and experimental evidence on multimodal neuroimaging in SAE, with emphasis on pathophysiological interpretation and translational relevance.

## 2 Pathophysiological mechanisms

The pathophysiology of SAE is multifactorial, reflecting the convergence of systemic inflammation, BBB dysfunction, microcirculatory failure, metabolic stress, and neurotransmitter imbalance (Czempik et al., 2020; Dumbuya et al., 2023).

First, Sepsis-induced inflammatory signaling triggers a surge of pro-inflammatory cytokines (e.g., TNF-α, IL-1β, IL-6), which induce neuroinflammation (Sonneville et al., 2023) by activating microglia and astrocytes and amplifying downstream inflammatory mediators and reactive oxygen species (ROS). In parallel, inflammatory endothelial injury increases BBB permeability, facilitating fluid extravasation and contributing to vasogenic and cytotoxic cerebral edema (Dumbuya et al., 2023). The imbalance between these two types of edema (vasogenic and cytotoxic) exacerbates brain injury by increasing intracranial pressure and disrupting neuronal function.

Microcirculatory dysfunction, caused by endothelial injury and coagulation disorders, further worsens the condition by reducing cerebral perfusion and increasing the risk of ischemic and hypoxic damage. Under sustained ischemic and hypoxic conditions, mitochondrial function is compromised, leading to reduced adenosine triphosphate (ATP) production, disrupted neuronal energy metabolism, and further exacerbation of neurological dysfunction (Gofton and Young, 2012; Catarina et al., 2021; Zhao et al., 2021).

The excitatory and inhibitory neurotransmitter systems are also disturbed in SAE. Glutamate dysregulation, in particular, plays a central role in the pathogenesis of SAE. Excessive glutamate release and impaired reuptake promote excitotoxicity, which leads to neuronal damage and apoptosis. On the other hand, disruptions in the GABAergic system contribute to synaptic signaling disturbances that play a role in the cognitive and behavioral deficits observed in SAE.

In summary, the pathophysiology of SAE involves complex interactions between inflammatory mediators, BBB disruption, and metabolic disturbances, leading to neuronal injury and dysfunction. Key cytokines such as TNF-α, IL-1β, and IL-6 initiate a cascade of events that include glial activation, ROS production, excitotoxicity, and neuroinflammation, all of which contribute to the observed clinical manifestations of SAE. These inflammatory pathways underlie the functional and structural changes seen in SAE, which include neuronal damage, network dysfunction, and, ultimately, long-term cognitive impairment.

**FIGURE 1:**
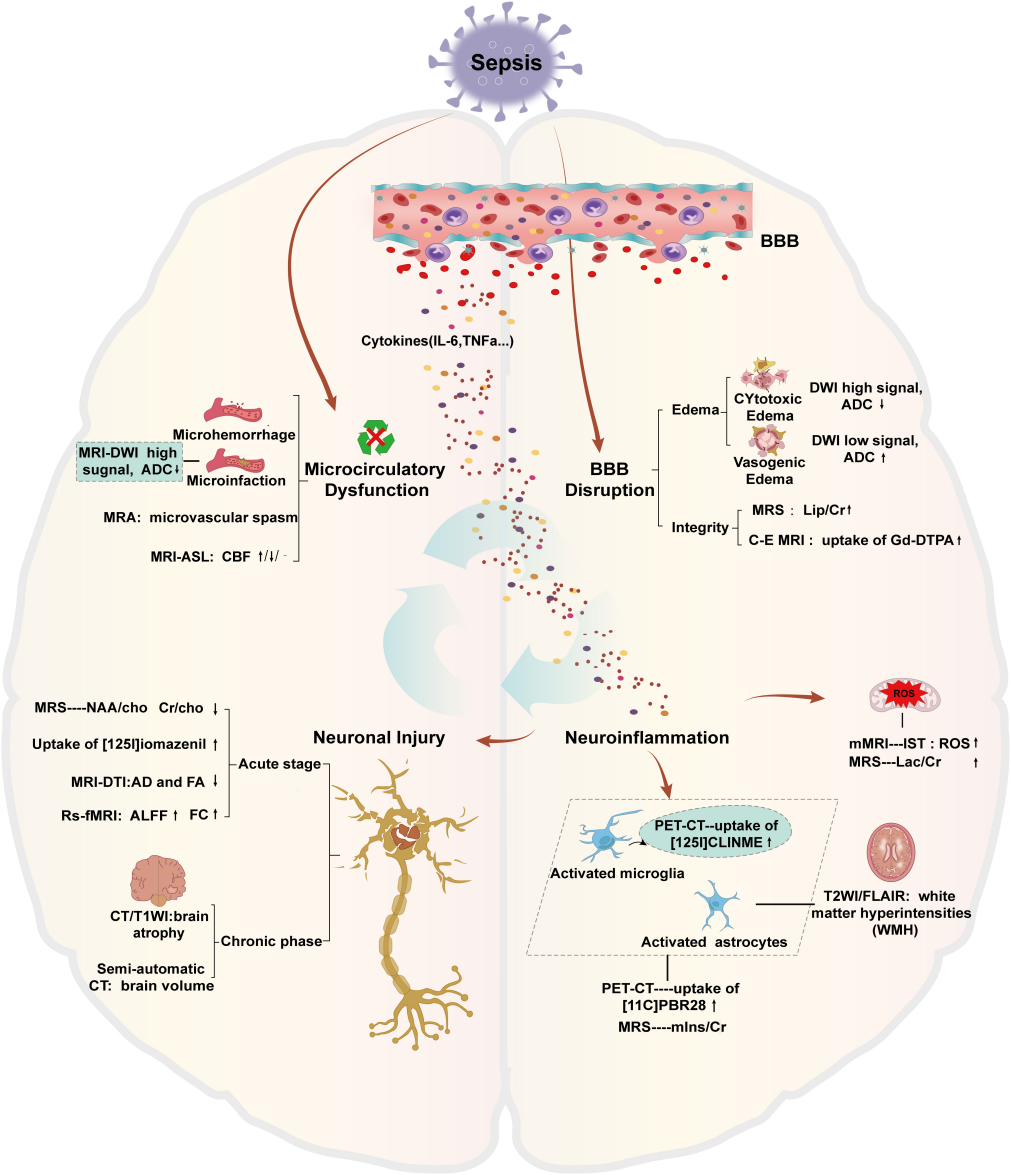
Proposed pathophysiological mechanisms of sepsis-associated encephalopathy and corresponding neuroimaging findings. Schematic overview of the major pathophysiological pathways involved in sepsis-associated encephalopathy (SAE) and their representative neuroimaging correlates. Sepsis-related systemic inflammation and circulating cytokines contribute to microcirculatory dysfunction and blood–brain barrier (BBB) disruption, which further promote neuronal injury and neuroinflammation. Microcirculatory impairment may lead to microhemorrhage, microinfarction, vasospasm, and cerebral blood flow (CBF) abnormalities (e.g., on DWI, MRA, and ASL). BBB disruption is associated with edema formation (cytotoxic and vasogenic edema) and altered BBB integrity, reflected by DWI/ADC changes, contrast-enhanced MRI, and metabolic alterations on MRS. Neuronal injury is characterized by oxidative stress, mitochondrial dysfunction, and acute-to-chronic structural/functional damage, with corresponding abnormalities on mMRI, MRS, DTI, rs-fMRI, CT, and conventional MRI. Neuroinflammation involves activation of microglia and astrocytes and may be detected by PET tracers and MRI white matter hyperintensities. Together, these findings illustrate a dynamic and interconnected imaging spectrum across the course of SAE.

These pathophysiological processes can be captured at different stages of SAE through multimodal imaging, which reflects a continuum of changes—from BBB disruption and microcirculatory dysfunction to metabolic and network disturbances (Figure 1).

## 3 Current diagnostic status

There is currently no unified diagnostic standard for SAE. In clinical practice, diagnosis relies on a comprehensive assessment of neurological manifestations, laboratory findings, and ancillary examinations, and remains largely a diagnosis of exclusion (Iacobone et al., 2009; Dumbuya et al., 2023). Recognition is further complicated by common ICU confounders, including sedation, analgesia, fever, mechanical ventilation, and systemic metabolic disturbances (Gofton and Young, 2012; Dumbuya et al., 2023). In addition, overlap with other acute encephalopathies in critically ill patients may further complicate interpretation, particularly when clinical or neuroimaging findings are non-specific.

Delirium is a common early manifestation and can be screened using tools such as the Confusion Assessment Method for the ICU (CAM-ICU; Ely et al., 2001) and the Intensive Care Delirium Screening Checklist (ICDSC; Bergeron et al., 2001); however, these instruments have limited specificity for SAE (Gofton and Young, 2012). Serum biomarkers such as neuron-specific enolase (NSE) and S100 calcium-binding protein B (S100B) may suggest neuronal injury in septic patients, but their limited sensitivity and specificity restrict their utility for distinguishing SAE from other encephalopathies (Nguyen et al., 2006; Piazza et al., 2007; Hsu et al., 2008). Electroencephalography (EEG) is highly sensitive in SAE, and abnormalities often precede overt clinical deterioration and broadly correlate with disease severity (Velissaris et al., 2018; Pantzaris et al., 2021). Typical findings include generalized slowing with predominant θ or δ activity, and in more severe cases, discontinuous background activity or burst-suppression patterns (Semmler et al., 2013; Hosokawa et al., 2014; Azabou et al., 2015; Berisavac et al., 2016; Pantzaris et al., 2021). However, EEG abnormalities remain non-specific and are strongly influenced by sedation and the ICU context, so they should be interpreted as supportive rather than diagnostic findings (Azabou et al., 2015; Berisavac et al., 2016; Ferlini et al., 2023).

Given these limitations, neuroimaging has become increasingly important as a complementary approach in SAE, both for excluding alternative diagnoses and for characterizing structural, diffusion, perfusion, metabolic, and functional brain abnormalities. The following sections therefore summarize the current applications and limitations of multimodal neuroimaging in SAE.

## 4 Applications and progress of imaging

Multimodal neuroimaging provides complementary structural, diffusion, perfusion, metabolic, molecular, and functional information in SAE. The following sections summarize the major imaging findings reported with each modality and their potential pathophysiological implications.

### 4.1 Computed tomography (CT)

Computed tomography is often the first-line examination due to its speed, accessibility, and low cost (Czempik et al., 2020). However, its diagnostic value in SAE is debated. Early studies, such as Jackson et al. (1985), reported low abnormality rates, with 11 of 12 SAE patients showing negative CT findings (Steiner, 2008). With larger cohorts and quantitative approaches, CT abnormalities have been reported more frequently. Sandquist et al. (2017) found that 63% of 149 septic children had abnormal final neuroimaging findings, including volume loss and CT density changes. CT is relatively sensitive to brain volume changes; in a retrospective cohort of septic ICU patients undergoing serial head CT, 87.5% exhibited brain volume reduction, with a mean change of −3.7% over a median of 31 days, supporting rapid atrophy (Nakae et al., 2021). Prospective data in elderly patients further support acute-phase brain volume reduction during sepsis (60%–79%) (Hosokawa et al., 2023). Case reports also show that CT may reveal diffuse brain edema in fulminant SAE, reflecting severe disease (Kondo et al., 2009).

In summary, while CT is primarily used to exclude hemorrhage or tumors, it can detect edema and atrophy, assisting in severity assessment.

### 4.2 Magnetic resonance imaging (MRI)

Magnetic resonance imaging is the core neuroimaging modality for evaluating SAE owing to its superior soft-tissue contrast and multiparametric capabilities. The following sequences provide complementary information on structural injury, edema, perfusion, metabolism, and network dysfunction.

#### 4.2.1 T1-weighted (T1WI) and T2-weighted imaging (T2WI)

T1-weighted is useful for depicting cerebral atrophy, such as sulcal widening and ventricular enlargement, whereas T2WI is more sensitive to edema and white matter lesions, which typically appear as parenchymal or white matter hyperintensities. For example, Becker et al. (2022) reported sepsis-related white matter signal abnormalities in 16 of 30 children with sepsis-related MRI abnormalities among 140 children with sepsis who underwent clinically indicated MRI, supporting white matter injury during sepsis.

#### 4.2.2 FLAIR sequence

Fluid-Attenuated Inversion Recovery (FLAIR) suppresses cerebrospinal fluid (CSF) signals, thereby enhancing lesion contrast.

(1) Non-contrast FLAIR: White matter hyperintensities (WMH) are common and are often distributed around Virchow–Robin spaces, supporting inflammation-associated BBB dysfunction; Lesions range from punctate to diffuse and correlate with shock duration and Glasgow Outcome Scores (GOS; Sharshar et al., 2007). FLAIR also clearly depicts vasogenic edema. FLAIR also clearly depicts vasogenic edema. Bartynski et al. (2006) reported vasogenic edema in patients with PRES associated with infection, sepsis, or shock, and most cases with available diffusion imaging did not show restricted diffusion, suggesting predominant vasogenic rather than cytotoxic edema in the acute phase. Animal studies similarly support early vasogenic edema (Griton et al., 2020). Some patients also show large vessel spasm and microvascular rarefaction on Magnetic Resonance Angiography (MRA), reflecting microcirculatory disturbance (Bartynski et al., 2006).

(2) Contrast-enhanced FLAIR: This sequence is superior for assessing BBB integrity. Leakage of contrast into the CSF appears as hyperintensity in sulci or ventricles, directly visualizing BBB breakdown.

#### 4.2.3 Diffusion-weighted imaging (DWI) and diffusion tensor imaging (DTI)

Diffusion-weighted imaging together with the ADC helps differentiate cytotoxic edema (DWI hyperintensity with reduced ADC) from vasogenic edema (Drake-Pérez et al., 2018; Zhao et al., 2021). Bozza et al. (2010) observed reduced ADC in the cortex and thalamus of CLP mice, consistent with cytotoxic edema, alongside T2 hyperintensity at the brain base suggesting vasogenic edema. In a small translational study, lateral hippocampal restricted diffusion was reported in patients with sepsis-induced delirium, suggesting a possible association with delirium (Zivkovic et al., 2015). However, timing matters; Rosengarten et al. (2008) found no significant ADC or T2 changes within 3.5 h in LPS mice, indicating that edema may not form immediately.

Diffusion-weighted imaging is also sensitive to ischemia. A prospective study found ischemic stroke in 29% of septic shock patients with acute neurologic changes (67% multiple), often accompanying leukoencephalopathy (Polito et al., 2013). A translational study provided evidence of diffuse axonal injury (DAI) and ischemia in SAE, aligning MRI findings with neuropathology (Ehler et al., 2017).

Diffusion tensor imaging (DTI) enables quantitative assessment of the rate and direction of water diffusion. In SAE, white matter injury may manifest as reduced fractional anisotropy (FA) and decreased axial diffusivity (AD), suggesting damage to white matter tract integrity. Dhaya et al. (2018) reported that, in LPS-treated rats, perfusion redistribution toward white matter-rich regions was altered, and horizontal water diffusion in the corpus callosum increased, supporting early white matter abnormalities. Using DTI, Zhu et al. (2022) also observed reduced FA and AD in the external capsule of experimental SAE, further supporting injury to white matter fiber tracts.

#### 4.2.4 Magnetic resonance spectroscopy (MRS)

Magnetic resonance spectroscopy enables non-invasive *in vivo* assessment of brain metabolism. In experimental SAE, studies have reported early metabolic abnormalities, including decreased NAA-containing ratios and increased mI/Cr, Glx/Cr, Lip/Cr, and Lac/Cr, although the exact pattern varies across models and time points (Bozza et al., 2010; Wen et al., 2017; Liu et al., 2024). Wen et al. (2017) further showed that decreased NAA/Cr was associated with hippocampal apoptosis. Although most studies show broadly concordant results, Towner et al. (2018) reported no decreases in NAA/Cho or Cr/Cho at 24 h in an LPS rat model, whereas decreases became evident at later time points, suggesting model- and time-dependent variability. Overall, MRS appears sensitive to early inflammatory and metabolic changes in experimental SAE (Liu et al., 2024). However, long acquisition times limit its use in unstable patients.

#### 4.2.5 Functional MRI (fMRI)

Resting-state functional MRI (rs-fMRI) measures spontaneous neuronal activity through blood oxygen level–dependent (BOLD) signals, and ALFF is a commonly used metric (Zou et al., 2008). In an rs-fMRI study, Li et al. (2022) reported increased ALFF in the hippocampus of CLP-induced SAE rats, particularly in the right CA1 region, suggesting altered spontaneous neuronal activity. Yao et al. (2022) further showed increased functional connectivity (FC) between the hippocampus and thalamus in SAE rats, and this enhanced connectivity correlated negatively with behavioral performance.

In an LPS-exposed rat model, Ji et al. (2018) observed increased FC between the retrosplenial cortex and medial prefrontal cortex, providing additional evidence of network-level connectivity abnormalities during sepsis-related brain dysfunction. Interventions like milk fat globule–EGF factor 8 (MFGE8) have been shown to reverse these ALFF elevations and improve survival (Li et al., 2024). Collectively, rs-fMRI–derived FC and ALFF alterations may provide clinically relevant insights for SAE diagnosis and for identifying candidate treatment targets.

#### 4.2.6 Arterial spin labeling (ASL)

Arterial Spin Labeling (ASL) is a perfusion imaging technique capable of assessing cerebral blood flow (CBF) without the need for exogenous contrast agents (Muir, 2018). Experimental studies have shown early hyperperfusion in both LPS-induced and CLP models. In an LPS rat model, Towner et al. (2018) detected increased cortical and thalamic rCBF at 24 h, followed by reduced cortical and hippocampal rCBF at later time points, while increased Gd-DTPA uptake was also observed in several brain regions. In a CLP mouse model, Bozza et al. (2010) likewise observed early increases in CBF in the cortex and thalamus. Another study reported region-selective hyperperfusion in white matter-rich regions, particularly the corpus callosum, rather than diffuse cortical hyperperfusion (Dhaya et al., 2018; Griton et al., 2020). These discrepancies may reflect differences in experimental model, imaging time window, and the brain regions analyzed.

In clinical settings, Masse et al. (2018) reported that vasopressor-dependent septic patients had 62% higher global CBF than sedated control subjects, and that CBF did not differ between a target MAP of 65 mmHg and ≥75 mmHg. Additionally, MRI studies by Orhun et al. (2019) and Sharshar et al. (2007) identified acute white matter lesions and perivascular-predominant abnormalities in sepsis-associated brain dysfunction, supporting the relevance of perfusion and BBB-related mechanisms. Collectively, these findings indicate significant alterations in cerebral perfusion during sepsis, with early hyperperfusion observed in experimental models and in a small clinical ASL study. These observations support a possible link between perfusion dysregulation, BBB disruption, and subsequent brain injury. In principle, ASL may help assess cerebrovascular autoregulation when interpreted together with hemodynamic variables such as MAP.

#### 4.2.7 Molecular MRI (mMRI)

Molecular magnetic resonance imaging (mMRI), combined with immuno-spin trapping (IST), can be used to detect free radicals generated by oxidative stress in experimental SAE (Towner et al., 2013). Increased radical-related signals have been detected in multiple brain regions in animal models (Catalão et al., 2017; Wu et al., 2019). The spin-trapping compound 5,5-dimethyl-1-pyrroline N-oxide (DMPO) captures free radicals, which are then detected using anti-DMPO probes. Under physiological conditions, anti-DMPO probes do not readily enter brain parenchyma, whereas in septic encephalopathy models, probe distribution within the brain becomes detectable, consistent with BBB disruption (Towner et al., 2018). Consequently, areas of mMRI signal enhancement may help localize radical-related brain injury *in vivo*.

Towner et al. (2013) first reported elevated free radical-related signals in the brain, liver, and lungs of CLP mice, with *in vivo* brain signal changes detected after 6 h of spin trapping and probe administration. Significant increases in DMPO-trapped radical signals were also detected at 24 h and 1 week following LPS administration (Towner et al., 2018). At present, mMRI remains limited to experimental studies but provides a unique approach for visualizing oxidative stress-related brain injury in SAE.

### 4.3 Positron emission tomography (PET)

Positron Emission Tomography (PET) uses specific molecular probes to achieve functional imaging. Combined with CT, it can localize brain abnormalities and help reveal pathological processes relevant to SAE, such as neuroinflammation and neuronal injury (Basu et al., 2011).

Neuroinflammation is one of the core pathological mechanisms of SAE. As a marker of activated microglia and astrocytes, translocator protein (TSPO) and its PET tracers have been used to evaluate neuroinflammation in experimental SAE. Giridharan et al. (2022) used [11C]-PBR28 and found increased tracer uptake at 24 h and 10 days after CLP surgery, supporting persistent glial activation and neuroinflammation.

In addition, [18F]-FDG PET can be used to assess brain glucose metabolism. Preclinical studies suggest that cerebral glucose metabolism in SAE may show time-dependent changes, with early increases in some models and decreased cortical metabolism in others. Szöllõsi et al. (2018) reported increased [18F]-FDG uptake at an early time point after LPS challenge, consistent with increased metabolic demand during early neuroinflammation. In contrast, Semmler et al. (2008) found that glucose uptake decreased in all analyzed neocortical regions, whereas uptake in the hippocampus and striatum remained unchanged. Zhu et al. (2022) further suggested that dynamic PET parameters may be more sensitive than static SUV measures for detecting early metabolic changes in experimental SAE. Animal studies also suggest that sepsis-associated brain dysfunction is accompanied by neurotransmitter abnormalities. Animal work summarized in prior reviews has also suggested increased GABA_A receptor-binding density in the forebrain, although this remains difficult to interpret mechanistically (Freund et al., 1985; Stubbs et al., 2013). These findings deepen the understanding of SAE pathology and provide potential targets for diagnosis and treatment. At present, PET ligands for neurotransmitter systems are available in other neurological settings, but their application in SAE remains largely exploratory, and studies directly linking neurotransmitter imaging to cognitive impairment in SAE are still lacking (Nikolaus et al., 2011; Walker and Rodda, 2011; Stubbs et al., 2013; Tables 1, 2).

**TABLE 1:** Preclinical neuroimaging studies in SAE: detailed methodologies and mechanisms.

Study	Model and sample size	Imaging sequences and endpoints	Key imaging findings (time post-sepsis)	Pathological correlates and mechanisms
Li et al., 2024	Rat, CLP (*n* = 60) Intervention: MFGE8	fMRI (rs-fMRI, ALFF) T2-MRI	7 d: CLP: ALFF in right CA3 MFGE8: ALFF in right CA1/CA3/DG ↓ T2-MRI: hippocampal hyperintensity after CLP; MFGE8 Group: Reversed ALFF/FC elevations; Prevented NAA decline.	Network and Excitotoxicity: Neuroinflammation drives network hyper-connectivity (ALFF/FC↑) and glutamate excitotoxicity. MFGE8 acts as an anti-inflammatory antagonist to rescue function.
Liu et al., 2024	Rat, CLP (*n* = 36) Acute	MRS: NAA, Lac, Lip, mIns Other: Histology	12h: Lac/Cr↑, Lip/Cr↑, mIns/Cr↑, NAA/Cr↓, Glx/Cr↑	Sepsis triggers simultaneous mitochondrial dysfunction (Lactate), membrane breakdown (Lipids), and glial activation, correlating with rapid neuronal apoptosis.
Yao et al., 2022	Rat, SAE Behavioral correlation	fMRI: rs-fMRI (FC analysis) Other: Emotional tests	Post-SAE: FC↑ between Hippocampus and Thalamus/BNST/Cortex. FC correlates with behavioral/emotional deficits.	Hippocampal network hyper-connectivity may contribute to behavioral and emotional dysfunction in SAE.
Zhu et al., 2022	Mouse, LPS (*n* = 12), 6–24 h	Dynamic [18F]-FDG PET/CT; CE-MRI; DTI MRI: DTI (FA, AD)	6 h: SUV↑, k4↑, Ki↓ 12 h: CE-MRI: T1 signal↑ 12–24h: FA↓ and AD↓ in external capsule.	Dynamic PET parameters (k4, Ki) detect early metabolic dysregulation preceding structural injury. CE-MRI supported BBB permeability increase, and DTI supported early white matter injury.
Giridharan et al., 2022	Rat, CLP (*n* = 3–6) Longitudinal study	PET: [11C] PBR28 (TSPO)	24 h/10 d: ↑ TSPO uptake in prefrontal cortex and hippocampus	TSPO upregulation was associated with microglial activation and astrocytosis, supported by IBA-1 and GFAP immunofluorescence.
Li et al., 2022	Rat, CLP (*n* = 24) Longitudinal study	fMRI: rs-fMRI (ALFF) MRS: Glx, mI, NAA Other: Behavior	7 d: ↑ ALFF in right CA ↑ Glx/Cr and ↑ mI/Cr ↓ NAA/Cho 14 d: ALFF decreased vs. 7 d (right CA1); Glx/Cr and mI/Cr remained elevated (vs. sham) NAA/Cho partially recovered	Hippocampal glial activation (mI↑) and metabolic/excitatory disturbance (Glx↑) were associated with behavioral impairment
Dhaya et al., 2018	Rat, LPS (*n* = 18) dynamic (0–150 min)	MRI: Phase-contrast MRI (PhC-MRI), ASL,DTI Other: IgG immunohistochemistry Target: Flow velocity	30–150 min: PhC-MRI: MCA flow velocity unchanged 90–150 min: ASL: CBF↑ in Corpus Callosum (Cortex unchanged). 150 min: DTI: ADC↑ parallel to callosal fibers/increased horizontal water diffusion	White matter-selective hyperperfusion may be related, at least in part, to BBB disruption and increased fiber-parallel diffusivity.
Ji et al., 2018	Rat, LPS Vs. PTSD (*n* = 48), 24 h Network analysis	fMRI: rs-fMRI (DMN FC) Other: Cognitive tests	24 h: LPS: ↑ FC (retrosplenial cortex–mPFC) PTSD: ↓ FC (retrosplenial cortex–visual cortex, insula, piriform cortex, sensory cortex) Distinct from PTSD pattern.	DMN Specificity: LPS-induced SAE showed a distinct pattern of DMN hyper-connectivity compared with PTSD, relevant to cognitive impairment.
Towner et al., 2018	Rat, LPS (*n* = 30) Longitudinal study	CE-MRI (Gd-DTPA) ASL (CBF) 1H-MRS (metabolism), mMRI (Free radicals)	24h: Gd-DTPA leakage; ASL: CBF↑; Free radicals↑ (mMRI). 1 w: Gd-DTPA leakage ↑/free radicals↑ 3w–12w: CBF↓; NAA/Cho↓, Cr/Cho↓.	Oxidative Stress and BBB: LPS induces free radical surge (mMRI) and BBB disruption leading to acute hyperperfusion followed by chronic metabolic crisis and hypoperfusion.
Wen et al., 2017	Rat, LPS (*n* = 35)	MRI: T2WI, ADC MRS: NAA, Lac, Cho Other: Serum NSE/S100beta	6 h: T2WI normal, MRS: NAA/Cr↓ 12–24 h: T2WI remained normal MRS: Further NAA/Cr↓vs. 6h	MRS shows early metabolic dysfunction (NAA/Cr decline) despite structurally normal T2WI at early time-points.
Bozza et al., 2010	Mouse, CLP (*n* = 25) Prospective study	MRI: T2WI, DWI/ADC, 1H-MRS Other: Behavior, Cytokines	6 h: T2WI: basal T2 hyperintensity (vasogenic edema); ADC↓ in the cortex and thalamus; MRS: NAA/Cho↓ 24 h: edema became more evident on T2WI	Early coexistence of vasogenic edema, cytotoxic edema, and neuronal metabolic injury in experimental SAE.
Semmler et al., 2008	Rat, LPS (*n* = 30) Prospective study	PET: [18F] FDG Other: regional CBF immunohistochemistry	24 h: [18F] FDG: neocortical glucose uptake↓; hippocampus, striatum, and thalamus preserved. cortical CBF↓, EEG alpha activity↓	Early neuroinflammation with impaired cortical metabolism and neuronal activity.
Rosengarten et al., 2008	Rat, LPS (*n* = 8 LPS/3 control)	MRI: T2WI, ADC Other: Physiological monitoring	3.5 h: No significant changes in T2 or ADC values despite endotoxic shock.	The short stimulus duration (3.5 h) is insufficient for the formation of detectable brain edema in this specific model.

Model: CLP, Cecal Ligation and Puncture; LPS, Lipopolysaccharide. RI: ADC, Apparent Diffusion Coefficient; ASL, Arterial Spin Labeling; DTI, Diffusion tensor imaging; DWI, Diffusion-weighted imaging; FA, fractional anisotropy; mMRI, Molecular MRI; MRS, Magnetic resonance spectroscopy; PHC-MRI, Phase-Contrast MRI. PET: FDG, Fluorodeoxyglucose; TSPO, translocator protein; SUV, Standardized Uptake Value. Functional: ALFF, Amplitude of Low-Frequency Fluctuations; DMN, Default Mode Network; FC, functional connectivity. Pathology: BNST, Bed Nucleus of the Stria Terminalis; GFAP, Glial Fibrillary Acidic Protein; Iba-1, Ionized calcium-binding adapter molecule 1; MFGE8, Milk Fat Globule-EGF Factor 8.

**TABLE 2:** Clinical neuroimaging studies in sepsis-associated encephalopathy (human).

Study	Design and population	Modality and timing	Key imaging findings	Pathological correlates and mechanisms
Orhun et al., 2022	Prospective study 156 adults with SAE	MRI (T1, T2, FLAIR, DWI) Timing: Median: 4 days after neurologic sign	PRES Pattern (8.9%): Frequently in atypical sites (frontal, corpus callosum, basal ganglia)	Endothelial dysfunction and hyperperfusion contribute to atypical PRES distribution.
Orhun et al., 2021	Prospective study 11 adults with SAE vs. 15 Primary headache controls.	MRI (T1, T2, FLAIR, DWI) Timing: Performed shortly after SAE diagnosis	Findings: T2/FLAIR showed white matter hyperintensities in 6 patients; additionally, global volume loss (atrophy) was noted in 3 patients	Biomarkers: Elevated sTREM2 and NfL levels reflect microglial activation and axonal loss.
Becker et al., 2022	Retrospective study 140 pediatric sepsis/septic shock patients	MRI (incl. SWI) Timing: Median: 9 days after sepsis onset	Sepsis-related lesions (21%): DWI/FLAIR showed white matter hyperintensities (53%);ischemia/infarct (30%); PRES (27%); SWI revealed microbleeds (20%).	Endothelial dysfunction and cytokine storm lead to heterogeneous injury patterns (edema, ischemia, hemorrhage). Sepsis-related MRI abnormalities associated with: ↑ ICU mortality (17% vs. 5%), ↑ new neurologic disability (32% vs. 11%),
Nakae et al., 2021	Retrospective study 48 septic adults with serial head CT	CT (semi-automated volumetry) Timing: First CT on admission; second CT at a median of 31 days after the first.	Brain Atrophy: Mean volume loss of 3.7% (−38.5 cm^3^) within 31 days;	Acute brain volume loss may be related to impaired cerebral blood flow, microthrombi formation, and neuroinflammation.
Orhun et al., 2019	Prospective study 93 SIBD patients	MRI (T1, T2, FLAIR, DWI/ADC); VBM Timing: mean 5.5 ± 5.4 days from neurologic signs to first MRI;	Normal:29% DWI/FLAIR: acute infarct 22.6%, WM lesions 16.1%, PRES 8.6%, encephalomalacia 7.5%, Atrophy 16.1%. VBM: reduced gray matter volumes	Abnormal MRI associated with higher disease severity; atrophy associated with increased mortality and elevated p-tau, supporting structural brain injury in SIBD.
Masse et al., 2018	Prospective study 10 septic patients (4 with chronic hypertension) vs. 12 controls (6 with chronic hypertension)	MRI-ASL (Perfusion) Timing: Performed at a mean of 1.5 days after ICU admission.	Hyperperfusion: Global CBF was 62% higher in the septic group compared to controls; CBF did not differ between MAP targets of 65 and ≥75 mmHg.	These findings support cerebral hyperperfusion and altered cerebrovascular regulation during vasopressor-dependent sepsis
Ehler et al., 2017	Translational study 13 septic shock;5 sepsis non-survivors; 14 septic rats. Vs. 3 human controls (non-sepsis deaths); 11 sham-operated rats.	Brain MRI (incl. DTI) + neuropathology Timing: Median:9 days after septic shock onset	FLAIR: WMH Pattern: punctiform (*n* = 3), patchy/confluent (*n* = 3), diffuse (*n* = 3) DWI: acute ischemic lesions in 3 patients.	MRI findings aligned with neuropathology, supporting DAI and ischemic injury
Sandquist et al., 2017	Retrospective study 389 pediatric sepsis (2004–2013)	CT/MRI Timing: Final imaging at median 157 days from first sepsis episode; early (0–14d) vs. late (>14d) analysis.	Abnormal final neuroimaging 63% (243/389) MRI: Among signal/attenuation abnormalities (*n* = 80): T2 hyperintensity *n* = 46, FLAIR hyperintensity *n* = 40, CT hypodensity *n* = 30; bilateral involvement in 87.5%.	Higher illness severity and oncologic diagnosis/organ transplantation were independently associated with abnormal neuroimaging.
Polito et al., 2013	Prospective study 71 septic shock patients with acute neurologic changes	MRI (incl. MRA) Timing: Performed at a median of 3 days after neurologic deterioration.	52% normal MRI 29% ischemic stroke (large/multiple/watershed infarcts) 21% leukoencephalopathy 8% mixed lesions	Ischemic stroke independently associated with DIC, and with ICU mortality. leukoencephalopathy discussed as possible BBB/endothelial process
Suchyta et al., 2009	Retrospective study 64 ICU patients with neurologic changes (sepsis subgroup 28%)	CT/MRI Timing: First brain scan at a mean of 4.3 ± 6.9 days after ICU admission.	Abnormal imaging 64% (41/64); atrophy (*n* = 26); WMH (*n* = 19); lesions (*n* = 17), edema (*n* = 8), focal hemorrhage (*n* = 7), encephalomalacia (*n* = 4).	Exploratory; frequent structural abnormalities reported, but no definitive imaging–outcome association established. not a pure SAE-specific cohort
Piazza et al., 2007	Retrospective study 21 ICU patients with severe sepsis	CT Timing: performed only when feasible (*n* = 5).	CT (*n* = 5): no cerebral lesions attributable to SAE reported.	S100B increase was non-specific and not correlated with encephalopathy severity
Sharshar et al., 2007	Prospective study 9 septic shock patients with brain dysfunction	MRI (incl. MRA) Timing: mean 1 day from neurologic signs to MRI; mean 7.8 days (median 7; range 2–24) after septic shock onset;	1. White matter lesions (7/9): –WMH FLAIR showed confluent hyperintensities in centrum semiovale, predominantly around Virchow-Robin spaces; – DWI hypointensity with increased ADC ↑ vasogenic edema 2. Multiple ischemic strokes (2/9) – DWI restriction with ADC reduction 3. Normal MRI: 2/9. 4. MRA: No large-vessel vasospasm	Endothelial leakage into perivascular spaces causes vasogenic edema; severity correlates with shock duration.
Bartynski et al., 2006	Retrospective study 25 PRES patients with infection/sepsis/shock (I/S/S)	CT/MRI (incl. MRA) Timing: PRES occurred at a mean of 6.7 days after infection onset; mostly within 2 weeks.	PRES: Vasogenic edema in parieto-occipital, temporal, and frontal white matter; DWI: largely normal (15/17). MRA (11/25): Diffuse vasospasm in severe hypertension; distal vessel pruning in normotensive patients; changes reversible on follow-up.	Endothelial dysfunction and autoregulatory disturbance contribute to vasogenic edema in infection/sepsis/shock-associated PRES.
Jackson et al., 1985	Retrospective study Autopsy 12 SAE cases	CT (*n* = 7) and Autopsy CT timing not specified/variable.	CT Negative: 6/7 scans were normal. Autopsy: Micro-abscesses found in 8/12 cases.	Early CT may be insensitive in septic encephalopathy, while structural brain lesions may still be present in severe cases.
Shindo et al., 2018	Case report;66-year-old Male Patients confirmed sepsis and diagnosed SAE	MRI (SWI) Timing: Day 15 and Day 60 after admission.	Day 15: FLAIR: WMH; SWI: microbleeds. Day 60: FLAIR partial resolution/atrophy; SWI microbleeds persist.	Microbleeds may indicate microvascular injury/BBB dysfunction; predictive value of acute MRI remains uncertain.
Garner et al., 2018	Case report; 19-year-old man with sepsis	CT/MRI Timing: CT on admission; MRI performed after seizure	Admission CT: No acute intracranial abnormalities. MRI (post-seizure): T2/FLAIR showed bilateral hyperintense signal in posterior parietal and occipital lobes	Sepsis-associated PRES with full clinical recovery after BP control.
Luitse et al., 2013	Case report;53-year-old Female with postoperative septic shock and deep coma ↑ diagnosed SAE	CT/MRI Timing: CT + CTA at day 5 post septic shock; CT day 9. MRI at 4 weeks post septic shock.	5 d: CT: diffuse white-matter hypodensity with global brain swelling; CTA normal; 9 d: no CT improvement 4 w: MRI: near-normal with few WM lesions, no infarcts.	Mechanism uncertain; authors cite possible BBB disruption and mitochondrial dysfunction in SAE; severe diffuse WM abnormality may be reversible.
Kondo et al., 2009	Case report;2 Children with Fulminant SAE	Serial CT/MRI/MRA Timing: Early CT within hours/day 1; MRI day 2 in one case; chronic MRI ∼day 43–106	1 d: CT: Diffuse brain edema 2 d: MRI (Pt2): DWI high signal in basal ganglia/subcortical white matter with low ADC Chronic MRI (days 43–106): FLAIR showed “cracking” lesions and diffuse white matter hyperintensity; MRA showed diminished intracranial arteries; venous sinus thrombosis.	Cytokine storm leads to extensive BBB breakdown, diffuse axonal injury (DAI), and venous thrombosis.
Finelli, 2004	Case report; 48-year-old woman with sepsis	MRI (T1, FLAIR, DWI) Timing: Performed on day 4 of hospitalization.	4 d: FLAIR hyperintensities in bilateral basal ganglia, cerebellum, brainstem, and temporal lobes DWI hyperintensity in basal ganglia No gadolinium enhancement	Autopsy: Multiple small infarcts in basal ganglia and cortex due to thrombotic microangiopathy.
Höllinger et al., 2000	Case report; 49-year-old Male with SAE complicated by cerebritis/abscess evolution	Serial CT/MRI Timing: Day 2 (CT), day 9 (MRI), day 21 (MRI), week 4 (CT).	2 d: CT: Non-enhancing hypodense white matter lesions 9 d: MRI: T2 hyperintense lesions DWI variably hyperintense. 21 d: MRI: Ring-enhancing lesions with concentric T2 structure (abscess formation); DWI signal regression. 4 w: CT: Ring enhancement, regressing hypodensities.	Imaging suggests rapid progression from cerebritis to abscess; early DWI hyperintensity (necrosis/cytotoxic edema) ↑ later vasogenic predominance.

AD, Axial Diffusivity; ADC, Apparent Diffusion Coefficient; ASL, Arterial Spin Labeling; CBF, Cerebral Blood Flow; DAI, Diffuse Axonal Injury; DTI, Diffusion tensor imaging; DWI, Diffusion-weighted imaging; FA, fractional anisotropy; FLAIR, Fluid-Attenuated Inversion Recovery; NfL, Neurofilament Light Chain; PRES, Posterior Reversible Encephalopathy Syndrome; SAE, Sepsis-Associated Encephalopathy; SIBD, Sepsis-Induced Brain Dysfunction; sTREM2, Soluble Triggering Receptor Expressed on Myeloid cells 2; SWI, Susceptibility-Weighted Imaging; VBM, Voxel-Based Morphometry; WMH, White Matter Hyperintensities.

## 5 Differential diagnosis

In neuroimaging, SAE is primarily characterized by diffuse, non-territorial abnormalities without evidence of direct CNS infection, which helps distinguish it from other neurological disorders. SAE imaging often reveals global white matter hyperintensities and diffuse signal changes on T2/FLAIR MRI, reflecting widespread blood–brain barrier dysfunction and neuroinflammation rather than focal lesions; structural imaging may be normal or show subtle changes, and contrast enhancement is uncommon in pure SAE. In contrast, acute ischemic stroke demonstrates focal DWI hyperintensity with restricted ADC values in specific vascular territories corresponding to an occluded artery, a pattern not seen in the diffuse encephalopathy of SAE. Similarly, viral encephalitis often produces asymmetric, regionally predominant T2/FLAIR hyperintensities (e.g., temporal lobes in HSV encephalitis) with variable contrast enhancement and associated mass effect, and autoimmune encephalitis typically affects limbic structures with segmental MRI abnormalities, both showing more regionally confined inflammation than the global changes of SAE. Metabolic encephalopathies present characteristic symmetric patterns—such as deep gray matter involvement in hypoglycemic or hepatic encephalopathy—and do not reflect the inflammatory BBB disruption central to SAE.

The use of clinical history and serial imaging over time helps differentiate these conditions, as SAE typically progresses with diffuse brain involvement and systemic sepsis rather than focal, localized injury.

## 6 Conclusion

This review summarizes the multimodal neuroimaging characteristics of SAE and their pathophysiological correlates. Common findings include white matter lesions, ischemic infarcts, edema, and atrophy, reflecting BBB disruption, microcirculatory dysfunction, metabolic disturbance, and neuroinflammation. SAE is typically characterized by diffuse white matter lesions and edema, detectable through techniques such as T2/FLAIR MRI and DWI, with reduced ADC values indicating both vasogenic and cytotoxic edema. In the acute phase, fMRI reveals altered connectivity, particularly in hippocampal regions, suggesting neuroinflammation and excitotoxicity. Mechanistically, these changes are driven by increased glutamate and ROS production, leading to neuronal injury and glial activation. BBB disruption plays a central role in exacerbating brain injury, as evidenced by increased Gd-DTPA leakage on contrast-enhanced MRI and elevated cerebral blood flow on ASL. Advanced imaging techniques such as DWI, MRS, fMRI, ASL, and PET provide further insights into edema patterns, metabolism, perfusion, network dysfunction, and molecular mechanisms, which help in understanding the full extent of SAE’s pathophysiology.

However, neuroimaging findings in SAE lack specificity, as they often overlap with those of other encephalopathies. Current limitations include heterogeneous study designs, variable imaging timing, limited specificity, and the logistical challenge of imaging critically ill patients. Future research should focus on refining multimodal imaging protocols, improving mechanistic validation, and strengthening the role of neuroimaging in early diagnosis, target identification, and therapeutic monitoring in SAE.
